# Classification of Student Leadership Profiles in Diverse Governance Settings: Insights from Pisa 2022

**DOI:** 10.3390/bs14080718

**Published:** 2024-08-16

**Authors:** Deniz Görgülü, Fatma Coşkun, Mete Sipahioğlu, Mustafa Demir

**Affiliations:** 1 Ministry of National Education Turkey, Konya 42070, Turkey; 2 Faculty of Education, Kahramanmaraş Sütçü İmam University, Kahramanmaraş 46050, Turkey; fatmacoskun@ksu.edu.tr; 3 International Relations Office, Samsun University, Samsun 55060, Turkey; mete.sipahioglu@samsun.edu.tr; 4 Faculty of Education, Bayburt University, Bayburt 69000, Turkey; mustafademir@bayburt.edu.tr

**Keywords:** student leadership, students’ leadership characteristics, latent class models

## Abstract

Student leadership prepares students for responsibilities, such as taking on specific tasks and assuming leadership roles in their future personal and professional lives. Developing students’ leadership profiles is among the important goals of educational systems aiming for future generations to take responsibility and advance their countries. With this perspective in mind, the PISA assessment includes items to measure students’ leadership behaviors. This study aims to extract student leadership profiles from the leadership-related items in the PISA 2022 application, using data from Cambodia, Peru, Paraguay, and Guatemala, which have different governance systems and cultural characteristics. The second purpose of the research is to determine the distribution of the identified leadership profiles in these countries and explain them in the context of governance and cultural characteristics. Latent class analysis was used to determine student leadership profiles. Accordingly, two-class and three-class latent models were found to be the most suitable models to explain student profiles. While the distinction between student profiles is more pronounced in the two-class model, the three-class model provides more detailed information about student profiles. In this respect, two-class and three-class latent models are reported comparatively. In the two-class latent model, students are labeled as the “Shy or Lack of Self-Confidence Group” and the “Active Leader or Influential Group”. In the three-class latent model, students are labeled as the “Moderate or Passive Leader Group”, the “Strong Leader or Influential Group”, and the “Avoidant or Leadership-Uncomfortable Group”. In both models, it is one of the striking findings that Cambodian students are in the low leadership profile, and Peruvian students are in the high leadership profile.

## 1. Introduction

There is no consensus on the definition and purpose of student leadership, as well as how it should be encouraged and labeled. However, in the literature, expressions such as participation, active citizenship, democratic school, and giving students a voice are used in relation to student leadership [1]. In this context, it can be said that student leadership should be taught to all students with a sense of social responsibility [2]. In this teaching process, learning leadership and developing leadership skills may require different learning methodologies than learning other subjects in a normal classroom environment, as it is different [3,4,5]. In this context, according to [6], students’ leadership competencies can be developed in two ways: through an on-the-job learning approach they learn through experience and a more intentional and developmental approach involving education, teaching, coaching, and feedback. In addition, implicit learning such as public awareness campaigns, political campaigns, informal learning, and socialization are also needed for the development of students’ leadership skills [7].

The values, thoughts, and behaviors that form the essence of leadership are social and interactive processes; therefore, they are culturally influenced. This fact causes leadership to be approached from different perspectives depending on cultural differences. For example, while leadership in Western societies is seen as based on a set of technical skills, in Chinese society, leadership is more seen as a process of influencing relationships and modeling desired behaviors [8]. There are many studies in the literature showing that leadership understanding differs in different cultures [9,10,11,12,13,14,15]. Based on the research on the leadership–culture axis, it can be argued that the leadership profiles of students continuing their education in different cultures may also differ. Furthermore, the implementation of a school-centered management strategy, known as democratization, has been found to have beneficial impacts on leadership [16]. Inside this particular framework, it is posited that the augmentation of democratization levels inside nations could potentially impact the leadership characteristics of individuals, particularly students, residing within said country. The results of some globally applied exams can be used to test the reality of this claim. PISA, TIMSS, PIRLS, and TALIS are among the exams applied globally [17,18,19,20]. PISA has emerged as a valid and reliable criterion for evaluating student performance, providing comparative data on educational outcomes among participating countries. It has gained significant influence on education policy and decision-making processes worldwide [21]. In the literature, there are studies focusing on the relation of PISA to countries’ education policies [22,23,24,25,26,27,28,29,30,31] and students’ competencies in certain areas (reading, scientific literacy, mathematical literacy, inquiry skill, financial literacy, etc.) [32,33,34,35,36,37,38,39,40]. However, no research examining the data on students’ leadership profiles included in PISA has been encountered. In this context, in our research, it will be examined whether there is a difference in the leadership profiles of students living in different countries, based on PISA results. For this, first, the reflections of the relationship between leadership and culture on individuals are discussed.

### Reflections of the Relationship between Leadership and Culture on Individuals

Although globalization has led to the convergence of many intercultural activities, cultural differences persist [41]. Societal culture has a major influence on the emergence of these differences. Societal culture imposes certain assumptions, ideas, traditions, and attitudes on the people living in that society, affecting their perceptions of reality and their behaviors within reality [42]. In this way, societal culture shapes acceptable and unacceptable behaviors in a given situation. At the same time, societal culture limits the leadership behaviors and characteristics accepted in that culture [43]. Summarizing the situation, [44] states that organizational and cultural factors influence the concept of leadership. Additionally, according to research, managers’ leadership styles and organizational work practices differ depending on the cultures in which they are applied and have various consequences on employee work outcomes [12,45,46,47,48].

The assumptions of role theory, defined by [49] as “a science concerned with the study of the characteristic behaviours of people in contexts and the processes that produce, explain, or are influenced by these behaviours”, can be used to demonstrate the relationship between leadership and culture. Role theory posits that expectations define roles [50]. According to this theory, leadership as a role is significantly influenced by certain ideals and leads to the preference of a particular leadership role in the workplace [51,52]. Additionally, role theory addresses the extent to which leadership behaviors are fixed or vary depending on values. The East–West dichotomy is one of the defined value categories [53]. Based on the assumptions of role theory, it can be said that leaders can redefine their roles by considering culturally significant symbolic values in their behaviors [9,54,55,56,57].

It is an undeniable reality that there are many similarities regarding the management processes of organizations in a globalized world. The literature focuses more on these common points rather than how leadership roles, behaviors, and styles can differ across different cultural or work contexts [58]. On the other hand, cultural fit seems to be overlooked in leadership-related theories and practices. This situation highlights a mono-cultural understanding while causing alienation, isolation, and disadvantages for indigenous and ethnic groups [59].

The evolution in leadership approaches also reveals the importance of culture in this regard. Accordingly, [60] analyzed contemporary approaches in the context of global leadership models under three sub-headings. These are the universal approach, which argues that leadership is a universal characteristic, the normative approach, which focuses on the characteristics and skills of global leaders, and the contingency approach, which rejects universal principles for effective leadership and recommends that leaders change their behaviors according to local characteristics. This approach emphasizes the role of situational variables and culture as contextual factors affecting leadership. In addition, this approach considers it necessary to consider the relationship between leader behavior and the situational environment, including cultural differences, in understanding effective leadership [61]. The Global Leadership and Organizational Behavior Effectiveness (GLOBE) project, as discussed by [11], investigates the impact of diverse cultures on leadership and work practices within various societies. The findings of the project align with the principles of the contingency approach and role theory. Its primary objective is to delve into leadership practices and behaviors across different cultural contexts, utilizing a comprehensive leadership survey comprising behavioral and attribute descriptors [62] The project uncovered notable variations in leadership preferences among different cultures [63]. Furthermore, the GLOBE project underscored the emergence of distinct leadership styles in diverse cultural settings [64]. 

At this point, the question of what should be the main issues to be considered in the leadership–culture relationship comes to mind. [65] argues that factors such as language, beliefs, values, religion, and social organizations cause cultural differences in the international perspective. In addition, it is stated that the good governance of countries, in other words, the understanding of governance followed in meeting social needs and providing services, is also effective in the understanding of leadership in that country [66]. In this context, it can be said that the management approaches of countries have a role in making the existence of different leadership practices more apparent. The existence in question can become dominant in the understanding of leadership as a cultural code. For example, while participatory and inclusive leadership practices are valued in a democratic management approach, organizational structures, discourses, speech, and communication within the hierarchy are given great importance [67]. In addition, in such managements, responsibility is distributed among members, empowering group members and enabling them to take part in decision-making processes [68,69,70,71,72]. In addition, in environments where democratic leadership is dominant, women’s leadership is supported for long-term socio-economic growth and great importance is attached to the diversification of the workforce and the empowerment of individuals [73]. These practices contribute to the positive development of individuals’ culture of democracy and democratic leadership understanding [74,75,76,77]. 

In societies where undemocratic leadership is dominant, situations such as concentration of power and authority in the hands of a few people, harsh decision making, violation of democratic norms and principles, absolute domination of subordinates, and disregard of subordinates’ contributions and suggestions are common [78,79,80,81,82]. However, it is also seen that information manipulations are used to create a positive image instead of transparency and honesty in communication [83]. In this context, absolute obedience and loyalty can be mentioned in conditions where undemocratic leadership is dominant [84,85]. In addition, the understanding of tolerating undemocratic behaviors is at a higher level in such environments [86,87,88]. In addition, it is seen that women leaders face many problems in undemocratic leadership and are dissuaded from being in leadership positions [89,90,91].

In addition to the theoretical context, existing studies in the literature show that culture and leadership are interrelated [92,93]; management style and cultural elements affect educational outcomes [94]. For example, in a study conducted in China [95], it was determined that students’ leadership skills developed in the form of loyalty to authority, collectivism, and respect for harmony. In a similar study conducted in Iran [92], it was noted that leadership profiles are influenced by cultural factors. It is well known that leadership style or leadership perception are influenced by local and cultural elements [96]. In the literature, there are many studies on school leadership [97,98,99] and instructional leadership [100,101]. However, there is no study examining the effect of the form of government and local culture on students’ leadership profiles. This has been the motivation for this study. Developing students’ leadership skills helps them to become individuals who guide others correctly, take responsibility, work hard, and make effective decisions in their business life or social roles in the following years [102]. Therefore, the leadership profiles of students are important indicators for the future of the society and the country in which they live. In addition to revealing the current situation of 15-year- old students at the international level, the PISA application also provides important information for the generation of countries that will soon be involved in business life. In this respect, the items related to leadership in the student questionnaires of the PISA 2022 application (items coded between ST305Q01JA–ST305Q10JA) are very important in terms of revealing whether students show different leadership profiles and examining whether these leadership profiles change according to the management style and cultural characteristics.

The socio-cultural fabrics and educational systems in Guatemala, Cambodia, Peru, and Paraguay each bear unique characteristics that shape the developmental contexts for students. In Guatemala, machismo culture emphasizes traditional gender roles that can influence leadership expectations [103]. The 36-year civil war also left scars, with marginalization of indigenous groups persisting [104].

Education in Guatemala faces challenges of decentralization, underfunding, and socioeconomic barriers. The system relies heavily on teacher-centered instruction with limited support structures for leadership development [105]. Cambodia’s education system has faced significant challenges since the Khmer Rouge regime, which decimated the teaching workforce and infrastructure [106] Despite progress in educational reconstruction, primary school leaders continue to grapple with issues stemming from the conflict’s legacy and broader developmental challenges [107]. The country’s teacher training programs, run by the government, have been rudimentary, with low salaries deterring well-qualified candidates [108].

Peru has shown progress in educational performance and socioeconomic development, but challenges remain. While student achievement levels are low, significant improvements were observed between 2007 and 2010, though progress has slowed recently [109]. Educational policies have been influenced by political changes, with periods of instability followed by increased coherence and leadership from 2011 to 2016 [110]. Despite progress, disparities in student achievement based on factors such as gender, location, school management, and socioeconomic status have intensified [109].

Paraguay faces challenges in implementing educational reforms, particularly in the areas of distributed leadership and bilingual education. Despite efforts to involve teachers in peer evaluation through programs like peer assistance and review (PAR), hierarchical norms, and implementation difficulties hinder the distribution of leadership responsibilities [111]. The National Plan for Educational Transformation 2030 aims to improve teacher performance evaluation and professional development, but obstacles remain in promoting innovation and collaborative work among educators [112].

These national distinctions hold implications for nurturing students’ leadership dispositions within each cultural-educational milieu. Particularly through middle adolescence covered by PISA, schools represent formative developmental settings that may variably foster certain leadership traits according to the priorities of each system. The following analysis considers how systemic conditioning may be reflected in student cohorts’ exhibited profiles.

In order to examine the leadership profiles of students, the data of Guatemala, Cambodia, Peru, and Paraguay of the PISA 2022 application were analyzed using latent class analysis (LCA). The reason why latent class analysis is preferred in examining students’ leadership profiles is that it is a person-oriented approach [113] and does not require assumptions such as sampling normality and homogeneity of variances [114]. LCA is a statistical method used to identify subgroups of a large population through a set of indicators [115]. In the selection of the countries included in the research sample, attention was paid to the fact that they represent different situations in terms of culture and management style variables that are expected to affect the distribution of student leadership profiles.

To summarize, the aim of this research is to examine the leadership profiles of students through the PISA 2022 data. In line with this aim, answers to the following questions are sought:

1. According to the PISA 2022 data, do students have different leadership profiles?
2. Do the leadership profiles exhibited by students vary between countries with different administrative styles and cultures?

## 2. Method

### 2.1. Sample

The PISA 2022 assessment formed the main data for this study. The analyses of the research were carried out on a total of 22,521 students from four countries, namely Guatemala, Cambodia, Paraguay, and Peru, which participated in the PISA 2022 assessment. In terms of their distribution in the sample, Guatemala accounts for 23% (5190), Cambodia 23.4% (5279), Paraguay 22.6% (5084), and Peru 30.9% (6968). A total of 81 countries from different continents of the world participated in the PISA 2022 assessment [116]. A two-stage sampling method was used in the sample selection for the countries participating in the PISA 2022 assessment [117]. Within the scope of this research, the countries of Guatemala, Cambodia, Paraguay, and Peru, which have different cultural and administrative understandings, were selected to determine the leadership profiles of students.

### 2.2. Data Collection Tools

The analyses of this research were carried out using the data from the student questionnaires of the PISA 2022 assessment. When examining the PISA 2022 reports, it was observed that the student questionnaires section included several items related to students’ leadership characteristics [118]. The items included in the student questionnaires within the scope of the PISA 2022 assessment inquire about the students’ degree of participation in relevant items that are indicators of the “leadership” characteristic. These items were graded with the options “Strongly disagree (1), Disagree (2), Neither agree nor disagree (3), Agree (4), Strongly agree (5)”. Detailed information about the items included in the questionnaire is shown in Table 1.

As seen in Table 1, the first column shows the item codes, the second column shows the item content, and the third column shows the information about the item rating scales. The research data were downloaded from the official website [119] where the PISA results are published.

## 3. Findings

Firstly, within the scope of the study, based on the research data formed by the samples of Guatemala, Cambodia, Paraguay, and Peru through the implementation of PISA 2022, whether students exhibit multiple leadership profiles was examined. This process was carried out in line with the research question formulated as “Are there different leadership profiles among students according to PISA 2022 data?”. The latent class analysis (LCA) technique was employed to answer the research question. LCA is a technique commonly used to identify latent groups in large samples. This technique is based on grouping individuals with similar response patterns into the same class based on their response patterns to items. In this respect, the classes to which individuals belong are defined as latent variables and explained through observable variables [120]. Generally, latent class analysis begins with a single-class model where individuals do not differ in response patterns, and the number of classes is increased until the most appropriate model is determined [121]. Accordingly, starting from a single-class latent model, parameter estimations were conducted by repeating LCA for models up to a six-class latent model. The model fit criteria for models up to a six-class latent model based on the research data are presented in Table 2.

In Table 2, Bayesian information criterion (BIC), the number of parameters, classification error, and class sizes are compared for six different models. These pieces of information are crucial for determining the model that best fits the data. BIC, also known as the Schwarz information criterion, was introduced by Gideon E. Schwarz in a paper published in 1978. BIC is a criterion calculated based on the number of parameters in the model when evaluating model fit in LCA. It is preferred for making comparisons based on corrected probability according to the complexity of the model. Lower BIC values indicate a better fit of the model to the data [122]. In this table, we can observe that as the number of models increases, BIC values generally decrease. This indicates that more classes improve the model’s fit to the data, but the rate of decrease in BIC is also important. The number of parameters indicates the complexity of the model. The fundamental goal of data reduction methods such as LCA is to explain complex data structures with the simplest model consisting of fewer variables. Classification error is a measure of the model’s ability to assign individuals to classes correctly. A lower classification error indicates better performance of the model. Class sizes indicate the relative magnitude of each latent class in the dataset. This shows how prevalent a particular class is.

In light of these considerations, when scrutinizing the model fit criteria presented in Table 2, it is imperative to strike a balance between the Bayesian information criterion (BIC) values, classification error, and the interpretability of the model when determining the most suitable model. Typically, preference is accorded to a model exhibiting low BIC values alongside a reasonable classification error. Nevertheless, one must also weigh the model’s complexity and interpretability. In this instance, the three-class model may be deemed a prudent selection, owing to its notably low BIC value and relatively modest classification error. Moreover, it is discernible that the distribution of class sizes is reasonable. However, the ultimate decision-making authority lies within the domain of the social sciences [123], contingent upon additional factors such as the research inquiry, dataset characteristics, and interpretative nuances [122]. In light of these multifaceted considerations, it appears judicious to employ and report the three-class latent model. However, in this study, both the three-class and two-class models are reported to scrutinize the variation in leadership attributes within the sample cohort vis-à-vis the number of classes. The three-class model emerges as the most cogent option for the dataset. Thus, in comparison to the three-class model, the two-class model yields diminished differentiation among students and furnishes less substantive insights for the sample cohort. The rationale behind eschewing the selection of the four-class model for comparative analysis predominantly stems from its markedly elevated classification error and the failure of all class sizes to confer meaningful proportions.

**Figure 1:**
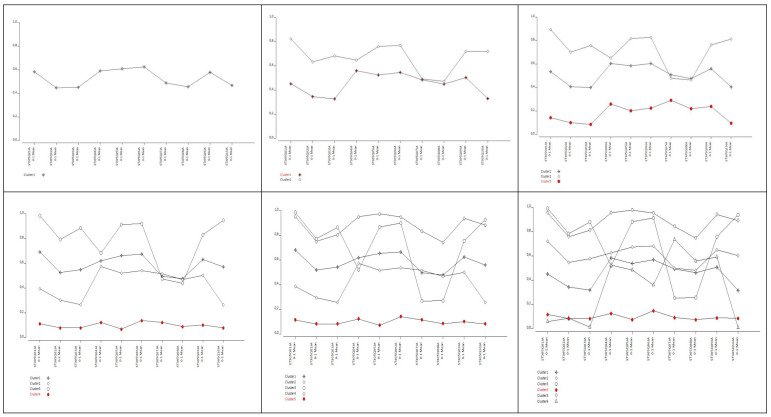
Profile plots of response probabilities for latent classes.

Consequently, both the two-class and three-class models evince superior classification accuracy and evince a judicious distribution concerning class sizes. Profile plots depicting response probabilities for all latent models up to six classes are depicted in Figure 1.

The plots seen in Figure 1 are one of the significant outputs of latent class analysis (LCA). Each line in these plots represents the characteristics of latent classes concerning different criteria, items, or, in other words, observable variables. Each point on the graph represents the mean response of classes for a specific criterion. In simpler terms, these plots illustrate profiles exhibited by individuals with similar response patterns in each graph. When observing Figure 1, it can be noticed that individuals with different profiles in the four-, five-, and six-class models are intertwined to the extent that they are almost indistinguishable from each other. This situation is also evident from the classification error value interpreted in the preceding paragraph referring to Table 2. As known, classification error is an indicator of the likelihood of errors in determining latent classes by the model. Accordingly, it can be inferred from Figure 1 that four-, five-, and six-class latent models are more complex and entail more errors in distinguishing participant profiles. Conversely, it can be said that two- and three-class latent models yield relatively better results. Interpretation of latent classes or participant profiles based on the graphs of latent models in Figure 1 is possible. However, to enhance the transparency of research findings and enable more detailed examinations, conditional response probabilities of observable variables for two- and three-class models are presented in Table 3.

According to the information in Table 3, for the two-class latent model, the first latent class (Cluster 1) encompasses individuals with low comfort with leadership roles and desire to lead, who tend to avoid sharing their opinions in group discussions. This group could be termed as “Reserved or Lack of Confidence Group”. The second latent class of the two-class model (Cluster 2) includes individuals who prefer taking on leadership roles and influencing others, generally being active in group discussions and enjoying taking initiative. This group could be labeled as “Active Leader or Influencer Group”.

For the three-class latent model, the first latent class (Cluster 1) may consist of individuals who respond neutrally or positively to leadership-related items but do not show a strong inclination towards leadership or influencing others. This group could be referred to as the “Moderate or Passive Leader Group”. The second class of the three-class model (Cluster 2) encompasses individuals who strongly enjoy assuming leadership roles and influencing others, actively participating in such roles. This group could be named as the “Strong Leader or Influencer Group”. The third class of the three-class model (Cluster 3) may include individuals who exhibit very low probabilities in leadership and influence- related aspects, often avoiding or feeling discomfort with such roles. This group could be designated as the “Avoidant or Discomfort with Leadership Group”.

When comparing the two- and three-class models, it is observed that the two-class model generally yields two broader groups representing specific behaviors or attitudes. On the other hand, in the three-class model, one or both of these general groups are further elaborated and subdivided into more specialized subgroups. This allows for the identification of more finely tuned groups with specific characteristics, aiding in a more detailed understanding of the behaviors or attitudes of these groups.

Secondly, within the scope of the research, using the PISA 2022 data, the distribution of students in the samples from Guatemala, Cambodia, Paraguay, and Peru was examined based on latent class analysis using both two- and three-class latent models. This process was conducted in line with the research question, which was formulated as “Do leadership profiles exhibited by students vary across countries with different governance styles and cultures?” The results are presented in Table 4.

When examining Table 4, it can be observed that in the two-class model, Cambodia mostly falls into the “Shy or Lack of Confidence Group”, whereas in the three-class model, it is more prevalent in the “Moderate or Passive Leader Group”. This suggests that students in Cambodia tend to exhibit more reserved or moderate tendencies in leadership-related situations. The distributions for Guatemala and Paraguay, in both countries, there is approximately equal distribution in the two-class model, while in the three-class model, the “Moderate or Passive Leader Group” and “Strong Leader or Influential Group” are more pronounced. This indicates a more diverse range of tendencies among individuals regarding leadership and influence in these countries. Looking at the distributions for Peru, it is observed that almost all students are in the “Active Leader or Influential Group” in the two-class model and in the “Strong Leader or Influential Group” in the three-class model. This suggests that students in Peru tend to be very active and influential in leadership matters.

These interpretations of the research findings imply that latent classes may vary across countries and that certain cultural or societal factors may influence these tendencies.

## 4. Discussion and Conclusions

In the study, the first latent class (Cluster 1) identified within the two-class model comprises individuals characterized by a low comfort level and desire to take on leadership roles, often avoiding sharing their own opinions in group discussions. This group could be labeled as the “Reserved or Lack of Self-Confidence Group”. The second latent class of the two-class model (Cluster 2) consists of individuals who prefer to assume leadership roles and influence others, typically actively participating in group discussions and enjoying taking initiative. This group could be termed the “Active Leaders or Influential Group”. For the three-class model, the first latent class (Cluster 1) may include individuals who respond neutrally or positively to items related to leadership but do not exhibit a strong inclination towards leadership or influence. They could be referred to as the “Moderate or Passive Leader Group”. The second class of the three-class model (Cluster 2) comprises individuals who strongly enjoy taking on leadership roles and influencing others, actively engaging in such roles. This group could be identified as the “Strong Leaders or Influential Group”. Finally, the third class of the three-class model (Cluster 3) may include individuals who exhibit very low probabilities in leadership and influence-related items, often avoiding or feeling discomfort with leadership roles. They could be labeled as the “Avoidant or Leadership-Discomfort Group”.

The finding that in Cambodia, students mostly belong to the “Reserved or Lack of Self-Confidence Group” in the two-class model and predominantly to the “Moderate or Passive Leader Group” in the three-class model suggests that Cambodian students tend to exhibit more reserved or moderate tendencies regarding leadership situations. Analyzing the distributions for Guatemala and Paraguay, while there is roughly equal distribution in the two-class model in both countries, the “Moderate or Passive Leader Group” and the “Strong Leaders or Influential Group” are more pronounced in the three-class model. This indicates a greater variety of tendencies in leadership and influence among individuals in these countries. Examining the distributions for Peru reveals that almost all students are part of the “Active Leaders or Influential Group” in the two-class model and the “Strong Leaders or Influential Group” in the three-class model. This suggests a tendency for students in Peru to be highly active and influential in leadership matters. These interpretations of research findings suggest that latent classes may vary across countries and that specific cultural or societal factors may influence these tendencies. This finding is consistent with the theoretical assumptions of role theory and the situational approach [12,124,125]. Additionally, these findings parallel the results of the GLOBE project, which highlights significant differences in leadership preferences across different cultures. Various studies within this framework emphasize similar outcomes [126,127,128,129,130].

In Guatemala, which has a long history of civil war and violence, the military held power for many years, and subsequently, the democratic governments that were established were also associated with incidents such as human rights violations and corruption. Despite democratically elected governments being in power today, the governance is characterized by a fragile structure due to the high likelihood of elites seizing control of the state administration [131]. This fragility can also be attributed to the country’s cultural fabric. 

Guatemala, composed of European descendants and the indigenous Maya people, is a multicultural society [132]. Due to this structure, the country is characterized as a multilingual nation with many ethnic groups [133]. When examining the main issues of Guatemala, a range of problems emerge, including a weak economy, a large youth population, state and gang-related violence, a lack of quality educational opportunities, and widespread poverty [134,135]. Guatemala is classified as an underdeveloped country due to high levels of poverty and inequality. Notably, the bilingual education services (Spanish and Maya languages) required by the multicultural structure present significant challenges [136]. Additionally, it is observed that the indigenous languages of Guatemala are not given the importance they deserve [137]. Furthermore, issues within the education system, such as inequalities and the necessity for children from poor and middle-class families to attend inadequate public schools, are prevalent [134]. According to data from the Ministry of Education in 2014, only 40% of sixth-grade students met national reading standards, and 44% met mathematics standards [138]. In summary, it can be said that Guatemala faces significant challenges in providing inclusive and quality education.

Cambodia is classified as a patrimonial or neo-patrimonial state, emphasizing the prevalence of patron-client relationships that extend from the top of the government in a complex pyramidal structure to distant villages. In other words, this country can be considered as a combination of dynastic governance and modern nation states [139]. In this context, it can be said that Cambodia’s democratization efforts are influenced by authoritarianism, political instability, and the legacy of past conflicts [140,141,142]. Furthermore, the internal conflicts in Cambodia’s historical development have negatively impacted the education system. This situation has led to difficulties in educational investments and teacher quality [63,143]. Additionally, an intensive curriculum, exam pressure, inadequate teacher salaries, corruption in education, rote learning, and oversight deficiencies have resulted in a high prevalence of private tutoring in Cambodia [144]. These conditions have also contributed to shortcomings in the Cambodian education system regarding equality, quality, and inclusivity—prerequisites for a democratic environment [145,146].

Another country governed by a presidential system is Paraguay [147]. As one of the last countries in South America to emerge from dictatorship, it is slowly transitioning towards democracy [148]. Despite stable growth in the country, there is a noticeable high level of inequality [149]. This situation can be attributed to the persistence of favoritism and corruption, despite significant changes implemented over the past 25 years [150]. Moreover, the eradication of poverty remains a distant goal. Currently, it seems unlikely that Paraguayans will be able to work in registered jobs with decent wages [151].

When examining Paraguay’s education system, it is evident that, similar to Guatemala, the use of Spanish and Guarani as official languages presents certain challenges in education. Guarani is an indigenous language spoken by 70.7% of the non-indigenous population. Despite numerous initiatives to integrate the Guarani language into the curriculum, none have proven effective. The majority of Guarani speakers are socioeconomically disadvantaged and reside in rural areas [148]. Furthermore, the increase in student numbers and average education duration in the country’s education system does not guarantee quality and equality in education. Particularly among indigenous peoples and children with disabilities, rural poverty and unequal access to health and protection services are significant factors preventing the full realization of the right to education. Statistics indicate that these inequalities limit educational opportunities. Additionally, due to low teacher performance, insufficient teaching and learning resources, an inappropriate curriculum, and weak educational management practices, the quality of education is far from the desired level [150].

On the other hand, Peru, governed by a presidential system and possessing a democratic system [152], is observed to be attempting to adopt neo-positivist ideals such as modernization and innovation with the support of the United States [153]. Peru’s ongoing democratic instability, corruption allegations, cultural migration, and multicultural structure contribute to the lack of national cohesion despite the presence of a national government for nearly two centuries [154,155]. The absence of decentralization, the neglect of the modern political class, and the provinces play a significant role in this lack of cohesion [156].

Peru is considered one of the Latin American countries that have invested the least in education over the past five years. Additionally, public investments in education in Peru are the responsibility of regional governments, leading to greater educational inequality among regions. However, it is noteworthy that school enrollment rates have reached between 80–85% in all regions, and dropout rates are lower, which is significant for education [157]. For the past 73 years, public education has been a fundamental area of Peru’s struggle for democratization. During this process, the groups that have strived the most to achieve the values of liberty, equality, and fraternity include women, indigenous peoples, people of African descent, Chinese people, Japanese people, Jewish people, rural communities, and those living in marginal areas of cities. Despite some improvements in education quality during the 2011–2016 period, the majority of illiterate people still come from poor rural areas and are indigenous women. This situation reveals that the level of illiteracy reflects the intersection of gender, ethnic, and social class inequalities in education [158]. Nevertheless, Peru has shown the most progress in reading among participating countries in the PISA exams since 2001. Additionally, the increase in enrollments at all levels of basic education and the steady rise in the education budget are positive developments for the Peruvian education system [159].

In conclusion, Guatemala, Cambodia, Paraguay, and Peru stand out as countries struggling with different governance systems and cultural structures. While Guatemala and Paraguay grapple with multilingual education systems and widespread poverty, Cambodia faces challenges in achieving equality and quality in education under the influence of patrimonial structures and past conflicts. Although Peru has made some progress in education during its democratization process, it still faces many challenges due to regional inequalities and low investment. All these countries encounter significant obstacles in providing inclusive and quality education in their education systems. In this respect, it can be said that the countries included in the research share similar characteristics.

The profiles outlined above regarding the countries provide some clues about the leadership profiles of students in the PISA results. Accordingly, individuals in the “Moderate or Passive Leader Group” in Cambodia, where dynastic rule still exists, may be associated with the country’s governance structure. On the other hand, the unequal structure in Paraguay and Guatemala, where democracy has not been fully established, may influence the distribution of students’ leadership profiles between the “Moderate or Passive Leader Group” and the “Strong Leader or Influential Group”. Additionally, in Peru, which is progressing towards becoming a democratic country with the support of the United States, it is believed that students’ placement in the “Active Leader or Influential Group” within their leadership profiles is not coincidental. In conclusion, it can be inferred that students in Cambodia, Guatemala, Paraguay, and Peru exhibit different leadership profiles, influenced by the diverse management styles and cultural structures of these countries.

In this study, we examined whether students exhibit different leadership profiles based on the PISA 2022 data and whether these profiles vary across countries with different governance styles and cultures. Regarding leadership profiles, we found that students in two-class models display different characteristics, such as “Shy or Lack of Confidence Group” and “Active Leader or Influential Group”, while in three-class models, they exhibit traits such as “Moderate or Passive Leader Group”, “Strong Leader or Influential Group”, and “Avoidant or Discomfort with Leadership Group”. Additionally, we identified variations in the leadership profiles of students in Guatemala, Cambodia, Paraguay, and Peru samples. These findings underscore the significance of the culture–leadership relationship and highlight the necessity of blending globally recognized leadership approaches with local characteristics on a global scale.

## 5. Practical Implications

Our findings have several important practical implications for education policy and practice:

- Tailored leadership-development programs: The identification of different leadership profiles among students (e.g., “Moderate or Passive Leader Group”, “Strong Leader or Influential Group”, and “Avoidant or Discomfort with Leadership Group”) suggests the need for tailored leadership development programs in schools. Educators can design interventions that address the specific needs of each group, helping to nurture leadership potential across all students.
- Cultural sensitivity in leadership education: The observed variations in leadership profiles across countries highlight the importance of culturally sensitive approaches to leadership education. Educational policymakers should consider local cultural contexts when developing leadership curricula, ensuring that leadership concepts are relevant and applicable to students’ lived experiences.
- Governance and leadership skills: The apparent influence of national governance styles on student leadership profiles suggests that civic education and leadership training should incorporate understanding of governance systems. This could help students develop leadership skills that are both globally relevant and locally applicable.
- Early intervention: Given that this study focused on 15-year-old students, the results underscore the importance of early intervention in leadership development. Schools and educational systems should consider implementing leadership programs from an earlier age to foster these skills throughout a student’s educational journey.
- Cross-cultural leadership exchange: The diversity of leadership profiles across countries presents an opportunity for cross-cultural leadership exchange programs. Such initiatives could broaden students’ understanding of leadership and prepare them for leadership roles in an increasingly globalized world.
- Teacher training: Our findings imply a need for specialized teacher training in leadership education. Teachers should be equipped with the skills to identify different leadership tendencies in students and to nurture these appropriately.
- Assessment of leadership skills: The use of PISA data in this study suggests that standardized assessments could incorporate more comprehensive measures of leadership skills. This could provide valuable data for ongoing research and policy development in student leadership.
- Inclusive leadership education: The identification of an “Avoidant or Discomfort with Leadership Group” highlights the need for inclusive leadership education that addresses the barriers some students face in developing leadership skills.

By implementing these practical implications, educational systems can work towards developing more effective, culturally relevant, and inclusive approaches to fostering leadership skills among students.

## Figures and Tables

**Table 1:** Item codes item content and response ranges for student leadership behaviour.

Item Code	Item Content	Response Range
ST305Q01JA	I am comfortable with taking the lead role in group.	1/2/3/4/5
ST305Q02JA	I know how to convince others to do what I want.	1/2/3/4/5
ST305Q03JA	I enjoy leading others.	1/2/3/4/5
ST305Q04JA	I keep my opinions to myself in group discussions.	1/2/3/4/5
ST305Q05JA	I speak up to others about things that matter to me.	1/2/3/4/5
ST305Q06JA	I take the initiative when working with my classmates.	1/2/3/4/5
ST305Q07JA	I wait for others to take a lead.	1/2/3/4/5
ST305Q08JA	I find it hard to influence people.	1/2/3/4/5
ST305Q09JA	I want to be in charge.	1/2/3/4/5
ST305Q10JA	I like to be a leader in my class.	1/2/3/4/5

**Table 2:** Model fit criteria for determining the number of classes.

Model	BIC *	Number of Parameters	Classification Error	Class Sizes
1 class	387,863.05	40	0.0000	-
2 classes	373,798.69	51	0.0748	0.65/0.35
3 classes	366,710.96	62	0.0604	0.73/0.21/0.06
4 classes	362,695.14	73	0.1155	0.50/0.38/0.08/0.04
5 classes	360,680.68	84	0.1191	0.51/0.36/0.06/0.03/0.04
6 classes	358,941.71	95	0.1321	0.45/0.41/0.05/0.03/0.03/0.03

* BIC (Bayesian information criterion).

**Table 3:** Variables for two- and three-class models.

Item	Item Level	2-Class Latent Model		3-Class Latent Model		
		Cluster 1 (0.6460)	Cluster 2 (0.3540)	Cluster 1 (0.7259)	Cluster 2 (0.2074)	Cluster 3 (0.0667)
(ST305Q01JA) I am comfortable with taking the role in a group.	Strongly disagree	0.9833	0.0167	0.5047	0.0031	0.4922
	Disagree	0.9672	0.0328	0.9040	0.0043	0.0917
	Neither agree nor disagree	0.8660	0.1340	0.9636	0.0194	0.0170
	Agree	0.5178	0.4822	0.7806	0.2115	0.0078
	Strongly agree	0.1302	0.8698	0.2414	0.7521	0.0065
(ST305Q02JA) I know how to convince others to do what I want.	Strongly disagree	0.8694	0.1306	0.5724	0.0754	0.3522
	Disagree	0.8424	0.1576	0.8897	0.0555	0.0548
	Neither agree nor disagree	0.6360	0.3640	0.8220	0.1695	0.0086
	Agree	0.4023	0.5977	0.6384	0.3576	0.0040
	Strongly agree	0.1516	0.8484	0.2547	0.7433	0.0019
(ST305Q03JA) I enjoy leading others.	Strongly disagree	0.9254	0.0746	0.5886	0.0342	0.3772
	Disagree	0.8925	0.1075	0.9140	0.0327	0.0534
	Neither agree nor disagree	0.6466	0.3534	0.8531	0.1421	0.0047
	Agree	0.3359	0.6641	0.6098	0.3870	0.0032
	Strongly agree	0.0776	0.9224	0.1528	0.8471	0.0001
(ST305Q04JA) I keep my opinions to myself in group discussions.	Strongly disagree	0.7484	0.2516	0.3820	0.2000	0.4180
	Disagree	0.7061	0.2939	0.7261	0.1737	0.1002
	Neither agree nor disagree	0.6840	0.3160	0.8093	0.1678	0.0229
	Agree	0.6524	0.3476	0.8084	0.1702	0.0215
	Strongly agree	0.4193	0.5807	0.5224	0.4487	0.0289
(ST305Q05JA) I speak up to others about things that matter to me.	Strongly disagree	0.9257	0.0743	0.4192	0.0347	0.5461
	Disagree	0.8840	0.1160	0.8647	0.0344	0.1009
	Neither agree nor disagree	0.7725	0.2275	0.8856	0.0878	0.0266
	Agree	0.5822	0.4178	0.7675	0.2182	0.0143
	Strongly agree	0.2859	0.7141	0.3962	0.5903	0.0135
(ST305Q06JA) I take the initiative when working with my classmates.	Strongly disagree	0.9548	0.0452	0.3939	0.0150	0.5911
	Disagree	0.9066	0.0934	0.8464	0.0245	0.1291
	Neither agree nor disagree	0.7746	0.2254	0.8903	0.0850	0.0246
	Agree	0.5934	0.4066	0.7735	0.2097	0.0168
	Strongly agree	0.2576	0.7424	0.3542	0.6296	0.0162
(ST305Q07JA) I wait for others to take the lead.	Strongly disagree	0.5933	0.4067	0.3930	0.3276	0.2794
	Disagree	0.6636	0.3364	0.7427	0.1938	0.0635
	Neither agree nor disagree	0.6570	0.3430	0.8117	0.1681	0.0202
	Agree	0.6729	0.3271	0.8042	0.1667	0.0291
	Strongly agree	0.5200	0.4800	0.5658	0.3610	0.0731
(ST305Q08JA) I find it hard to influence people.	Strongly disagree	0.6242	0.3758	0.4068	0.2976	0.2956
	Disagree	0.6797	0.3203	0.7628	0.1755	0.0616
	Neither agree nor disagree	0.6451	0.3549	0.7943	0.1849	0.0207
	Agree	0.6494	0.3506	0.7894	0.1885	0.0221
	Strongly agree	0.4975	0.5025	0.5732	0.3808	0.0461
(ST305Q09JA) I want to be in charge.	Strongly disagree	0.8779	0.1221	0.4557	0.0713	0.4730
	Disagree	0.8683	0.1317	0.8671	0.0527	0.0802
	Neither agree nor disagree	0.7061	0.2939	0.8356	0.1405	0.0239
	Agree	0.5642	0.4358	0.7478	0.2324	0.0197
	Strongly agree	0.3474	0.6526	0.4574	0.5164	0.0262
(ST305Q10JA) I like to be a leader in my class.	Strongly disagree	0.9553	0.0447	0.6232	0.0171	0.3597
	Disagree	0.9234	0.0766	0.9249	0.0156	0.0595
	Neither agree nor disagree	0.6702	0.3298	0.8903	0.1036	0.0060
	Agree	0.3192	0.6808	0.6013	0.3954	0.0033
	Strongly agree	0.0859	0.9141	0.1674	0.8291	0.0035

**Table 4:** Distribution probabilities of research countries in two- and three-class models.

Country	2-Class Latent Model		3-Class Latent Model		
	Cluster 1	Cluster 2	Cluster 1	Cluster 2	Cluster 3
Cambodia	0.8262	0.1738	0.8735	0.0595	0.0670
Guatemala	0.5763	0.4237	0.6504	0.2781	0.0715
Paraguay	0.5610	0.4390	0.6628	0.2691	0.0681
Peru	0.0004	0.9996	0.0028	0.9972	0.0000

## Data Availability

Data are contained within the article.
